# Modulation of Bronchial Epithelial Barrier Integrity by Low Molecular Weight Components from Birch Pollen

**DOI:** 10.3390/ijms25137374

**Published:** 2024-07-05

**Authors:** Srinidhi Sudharson, Tanja Kalic, Julia Eckl-Dorna, Nina Lengger, Heimo Breiteneder, Christine Hafner

**Affiliations:** 1 Department of Dermatology, University Hospital Sankt Poelten, Karl Landsteiner University of Health Sciences, 3100 Sankt Poelten, Austria; srinidhi.sudharson@kl.ac.at (S.S.); tanja.kalic@kl.ac.at (T.K.); 2 Institute of Pathophysiology and Allergy Research, Center for Pathophysiology, Infectiology and Immunology, Medical University of Vienna, 1090 Vienna, Austria; nina.lengger@meduniwien.ac.at (N.L.); heimo.breiteneder@meduniwien.ac.at (H.B.); 3 Department of Otorhinolaryngology, Medical University of Vienna, 1090 Vienna, Austria; julia.eckl-dorna@meduniwien.ac.at

**Keywords:** birch pollen, bronchial epithelial cells, cytokines, epithelial barrier integrity, low molecular weight components, transepithelial electrical resistance, xCELLigence

## Abstract

Pollen, in addition to allergens, comprise low molecular weight components (LMC) smaller than 3 kDa. Emerging evidence indicates the relevance of LMC in allergic immune responses. However, the interaction of birch pollen (BP)-derived LMC and epithelial cells has not been extensively studied. We investigated epithelial barrier modifications induced by exposure to BP LMC, using the human bronchial epithelial cell line 16HBE14o-. Epithelial cell monolayers were apically exposed to the major BP allergen Bet v 1, aqueous BP extract or BP-derived LMC. Barrier integrity after the treatments was monitored by measuring transepithelial electrical resistance at regular intervals and by using the xCELLigence Real-Time Cell Analysis system. The polarized release of cytokines 24 h following treatment was measured using a multiplex immunoassay. Epithelial barrier integrity was significantly enhanced upon exposure to BP LMC. Moreover, BP LMC induced the repair of papain-mediated epithelial barrier damage. The apical release of CCL5 and TNF-α was significantly reduced after exposure to BP LMC, while the basolateral release of IL-6 significantly increased. In conclusion, the results of our study demonstrate that BP-derived LMC modify the physical and immunological properties of bronchial epithelial cells and thus regulate airway epithelial barrier responses.

## 1. Introduction

The airway epithelium serves as a first line of defense against inhaled pathogens, pollutants and allergens. This protective barrier is composed of epithelial cells that possess a multitude of membrane receptors. These receptors interact directly with allergen sources, resulting in the release of inflammatory mediators such as chemokines, cytokines and alarmins [1,2,3]. In susceptible individuals, exposure to pollen can lead to allergic sensitization and the subsequent development of allergic inflammation [4,5]. This process involves an interplay between immune cells and the epithelial barrier, highlighting the importance of the airway epithelium not only as a physical barrier but also as an active member in immune regulation [3,5].

Birch pollen (BP) is a major elicitor of allergic rhinitis and asthma, particularly in temperate regions of Europe, North America and parts of Asia, affecting millions of people in these areas [6]. The key sensitizing BP allergen is Bet v 1, which belongs to the PR-10-like protein family [7]. The pollen matrix consists of diverse bioactive intrinsic molecules such as lipids, carbohydrates and other metabolites, which are co-delivered with the allergens [4,8,9]. Many of these molecules are low molecular weight components (LMC) that are smaller than 3 kDa and they have been investigated for their role in regulating immune responses. Pollen- and fish-derived LMC have been shown to modify epithelial properties and induce a polarized release of pro-inflammatory cytokines [9,10]. Adenosine, a nucleotide, constituted a major component in pollen-derived LMC, exhibiting a potential regulatory role in allergic inflammation [11,12]. A nasal challenge with BP-derived LMC in individuals with pollen allergies demonstrated an elevated local release of IL-8 and nasal symptoms [13]. In vitro studies have demonstrated that lipid mediators from pollen recruit and activate granulocytes, emphasizing their potential contribution to allergic inflammation [14,15,16].

It is essential to study the interactions between the epithelium and LMC to understand their role in allergic sensitization. Thus, the objective of this study was to investigate the barrier properties of bronchial epithelial cells upon treatment with BP-derived LMC. This was achieved by measuring transepithelial electrical resistance (TEER) to assess barrier function, monitoring barrier integrity using real-time cellular impedance assays, and quantifying the compartmentalized release of inflammatory mediators. Our findings indicate that BP-derived LMC significantly modify bronchial epithelial properties.

## 2. Results

### 2.1. BP LMC Increase Barrier Integrity in Bronchial Epithelial Cells

**Figure 1:**
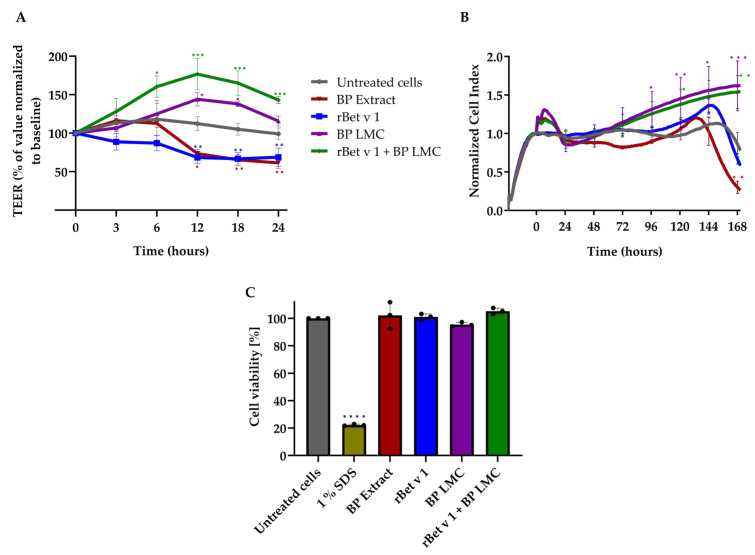
BP LMC increase barrier integrity. (**A**) Confluent polarized 16HBE14o- cell monolayers were apically treated with BP extract (250 μg/mL), rBet v 1 (100 μg/mL), BP LMC (25% *v*/*v*) or combination of rBet v 1 and BP LMC. Untreated monolayers were used as controls. TEER was monitored for 24 h following treatments. Percentage TEER represent TEER values normalized to the untreated monolayers at t = 0 h (baseline). Data were obtained from three independent experiments and are represented as mean % TEER ± SD. (**B**) The cell monolayers were exposed to BP extract (250 μg/mL), rBet v 1 (100 μg/mL), BP LMC (25% *v*/*v*) or a combination thereof, and the epithelial barrier integrity was monitored for 168 h following treatments using the xCELLigence Real-Time Cell Analysis system. The Cell Index values were normalized to the time point of treatment (t = 0 h) and are presented as Normalized Cell Index values. Data represent mean Normalized Cell Index ± SD from four experiments performed in triplicates. (**C**) Confluent cell monolayers were exposed to BP extract (250 μg/mL), rBet v 1 (100 μg/mL), BP LMC (25% *v*/*v*) or a combination thereof and cell viability was measured after 24 h. For reduced cell viability, 1% SDS was used as positive control. The percentage of viable cells was calculated by using untreated cells as reference. Data represent mean percentage cell viability ± SD from three experiments, and the asterisks above each bar indicate a statistically significant difference as compared to the viability of untreated cells. * *p* < 0.05, ** *p* < 0.01, *** *p* < 0.001, **** *p* < 0.0001.

The barrier integrity and permeability of 16HBE14o- cells was monitored by periodic TEER measurements up to 24 h after apical treatments as shown in Figure 1A. A significant increase in TEER was observed in cells exposed to BP LMC, peaking at 12 h with a 28% ± 8% increase in TEER in comparison to untreated cells, and persisting until 18 h. Exposure to the combination of recombinant (r) Bet v 1 and BP LMC significantly increased TEER as early as 6 h, up to 35% ± 15% when compared to untreated cells, and reached a peak after 12 h with a 57% ± 13% increase. In contrast, exposure to BP extract or rBet v 1 induced a significant decrease in TEER up to 35% ± 4% and 40% ± 5% after 12 h, and 38% ± 5% and 31% ± 8% after 24 h, respectively.

An impedance-based xCELLigence Real-Time Cell Analysis system was utilized as an additional approach to observe the barrier integrity of 16HBE14o- cells. After growing to confluency, the cells were exposed to BP extract, rBet v 1, BP LMC or a combination thereof, and the Cell Index (CI) was followed for 168 h (7 days) as illustrated in Figure 1B. Exposure to rBet v 1 did not result in any statistically significant differences compared to untreated cells, while treatment with BP extract led to a sharp decrease after 144 h (day 6), reaching a CI of 0.3 after 168 h (day 7). Exposure to BP LMC significantly enhanced epithelial barrier integrity in comparison to untreated cells with a first peak after 6 h, reaching a CI of 1.3, followed by a decrease to the level of untreated cells after 24 h. This was followed by a gradual increase in CI, reaching a value of 1.3 after 96 h (day 4) and of 1.6 after 168 h (day 7), respectively. The combined exposure of rBet v 1 and BP LMC demonstrated comparable CI values to those observed in the BP LMC treatment alone. Cell viability was not affected by the treatment with rBet v 1, BP extract or BP LMC as depicted in Figure 1C.

### 2.2. Effect of BP LMC on Papain-Damaged Epithelial Barrier

**Figure 2:**
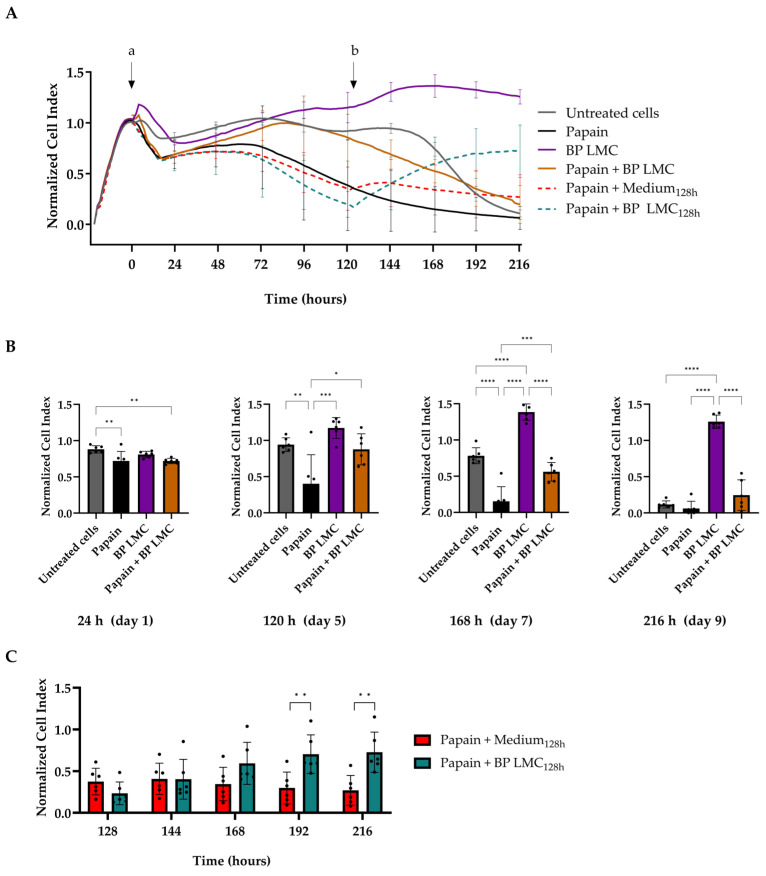
Time-lapse measurements of transepithelial impedance of bronchial epithelial cells. Monolayers of 16HBE14o- cells were exposed to BP LMC (25% *v*/*v*), papain (70 µg/mL) or a combination thereof at t = 0 h as indicated by the arrow (a). Epithelial barrier integrity was monitored for 216 h (9 days) using the xCELLigence Real-Time Cell Analysis system. After 128 h, the cells exposed to papain at t = 0 h were treated with BP LMC or medium (b) as indicated by the arrow. The Cell Index values were normalized to the time point of treatment (t = 0 h) and represented as Normalized Cell Index values. (**A**) Overview of the Normalized Cell Index values over the measured period of 216 h. Adjusted *p* values of comparisons between the Normalized Cell Index values after treatments are shown in Supplementary Data, Table S1. (**B**) Normalized Cell Index values at 24, 120, 168 and 216 h after exposure. (**C**) Normalized Cell Index values at 128, 144, 168, 192 and 216 h after exposure of papain-treated cells to BP LMC (or medium) at t = 128 h. Data represent mean Normalized Cell Index ± SD from six experiments performed in triplicates. Asterisks indicate a statistically significant difference between the compared groups. * *p* < 0.05, ** *p* < 0.01, *** *p* < 0.001, **** *p* < 0.0001.

Confluent cells were treated with papain to damage the epithelial barrier due its ability to mediate epithelial barrier injury [17,18]. The cellular impedance was monitored for 216 h (9 days) after exposures as shown in Figure 2A. The transepithelial impedance of cells exposed to BP LMC increased significantly at 144 h (day 6) with a CI value of 1.31 ± 0.1, compared to untreated cells with a CI value of 0.94 ± 0.05. Treatment with papain resulted in a statistically significant decline in barrier integrity between 120 h (day 5) and 168 h (day 7) (Figure 2B, Supplementary Data, Table S1). Simultaneous exposure to BP LMC and papain showed no significant differences in CI values in comparison to untreated cells (Figure 2A,B). However, the CI values of cells exposed simultaneously to BP LMC and papain increased significantly between 120 h (day 5) and 192 h (day 8) compared to cells exposed to papain only.

Subsequently, when the CI value of papain-treated cells was reduced by 68% ± 11% after 128 h (day 5), the cells were exposed to BP LMC. The impedance of these cells significantly increased after additional BP LMC exposure at 192 h (day 8) with a CI of 0.7 ± 0.23, persisting until 216 h (day 9) when compared to papain-treated cells (Figure 2C). This part of the experiment needed the exchange of 20% of the total medium volume to allow for the addition of BP LMC as described previously. Consequently, a direct comparison to all other conditions was not conducted.

### 2.3. BP LMC Influence Polarized Release of Cytokines

To analyze the epithelial immune response after treatment with the pollen components, the polarized release of cytokines 24 h after exposure was measured using multiplex immunoassays. The concentrations of 37 cytokines were measured. The concentrations of 15 cytokines were below the detection limit for all the treatments, and the concentrations of the 22 quantified cytokines are listed in Supplementary Data, Table S2.

**Figure 3:**
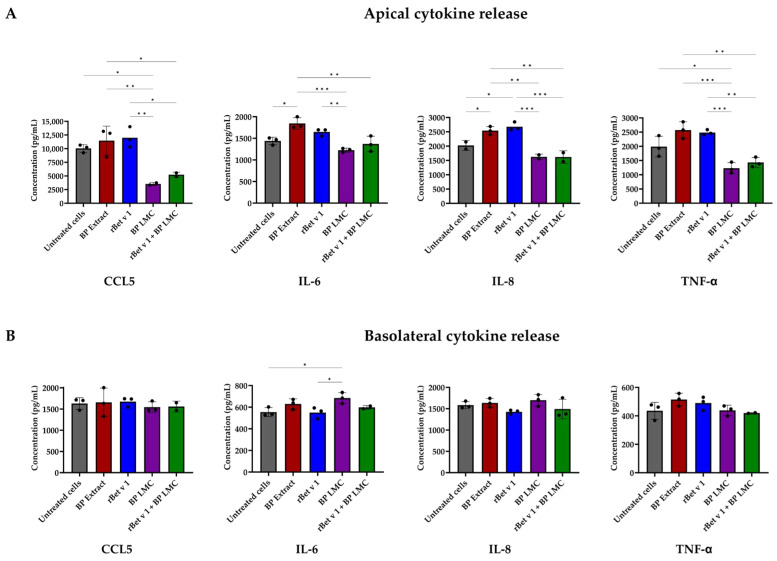
Quantification of compartmentalized release of mediators. Polarized 16HBE14o- cell monolayers were treated with BP extract (250 μg/mL), rBet v 1 (100 μg/mL), BP LMC (25% *v*/*v*) or a combination thereof. Untreated cells were used as control. Apical and basolateral supernatants were collected 24 h after treatments and the mediators were measured. Data represent results from three different experiments. Graphs depict mean cytokine concentrations ± SD released at (**A**) apical and (**B**) basolateral compartments respectively. Asterisks indicate a statistically significant difference between the compared groups. * *p* < 0.05, ** *p* < 0.01, *** *p* < 0.001.

A significant decrease in the apical secretion of CCL5 (*p* = 0.0172) and TNF-α (*p* = 0.0224) in comparison to untreated cells was observed after exposure to BP LMC, while a significant decrease in IL-6 (*p* = 0.0005, *p* = 0.0081) and IL-8 (*p* = 0.0023, *p* = 0.001) in comparison to BP extract or rBet v 1, respectively, was observed, as illustrated in Figure 3A. The combined treatment of rBet v 1 and BP LMC leads to a significant decrease in IL-6 (*p* = 0.0036) in comparison BP extract exposure and a decrease in CCL5 (*p* = 0.0215, *p* = 0.0137), IL-8 (*p* = 0.0022, *p* = 0.0009) and TNF-α (*p* = 0.0014, *p* = 0.0025) levels compared to BP extract or rBet v 1 exposure, respectively. In contrast, the basolateral release of IL-6 increased significantly after the exposure to BP LMC (*p* = 0.0422), while no statistically significant changes were observed in the basolateral secretion of the other quantified cytokines (Figure 3B).

The amount of each cytokine secreted to the apical and basolateral compartments and thereby the total amounts of cytokines released after exposure to BP LMC are visualized in Supplementary Data, Figure S1. A significant reduction in the overall secretion of cytokines IL-1ra (*p* = 0.0332), CCL26 (*p* = 0.0028) and IL-7 (*p* = 0.0084) was observed following BP LMC exposure. In contrast, a significant increase in CCL2 (*p* = 0.0358) was evident after exposure to BP LMC. The apical secretion of CCL5 decreased significantly without affecting the basolateral secretion (Figure 3 and Figure S1A). A noticeable elevation, though not significant, was observed in the basolateral levels of IFN-γ and IL-9 upon BP LMC exposure.

## 3. Discussion

Studies have indicated that LMC modify epithelial barrier properties [9,10] and can act as potential drivers of a Th2 immune response [11,13,19,20]. Pollen derived-LMC consist of a diverse array of molecules such as lipids [14,16], nucleosides [11] and flavonoids [21,22]. However, the influence of birch pollen-derived LMC on epithelial cells is not yet fully understood. In the current study, we have demonstrated that BP LMC modify bronchial epithelial properties by enhancing epithelial barrier integrity and modifying cytokine release.

Two different methods were employed to assess epithelial integrity after exposure to BP-derived components—TEER measurement and the xCELLigence system. With both methods, we observed an enhancement in epithelial barrier integrity upon exposure to BP LMC without affecting cell viability (Figure 1). We also tested the combined effects of the recombinant major BP allergen, rBet v 1, and BP LMC, and observed an increase in TEER, implying that rBet v 1 does not influence the effect of BP LMC. Nevertheless, further investigations are necessary to understand LMC–allergen interaction and to determine if LMC influence the transport of allergens across the epithelium.

In contrast to a previous study that demonstrated an increase in TEER upon exposure to BP aqueous extract [9], our findings indicated a significant decrease in TEER (Figure 1A) without affecting cell viability. Yet, our results are in line with those of the aforementioned study regarding LMC, where exposure to grass pollen-derived LMC also led to an increase in TEER [9]. Studies investigating LMC included not only pollen-derived LMC but also LMC derived from food allergen sources. One such study demonstrated that exposure to cod-derived LMC led to a decrease in TEER at 24 h while shark-derived LMC led to an increase in TEER [10]. These observations suggest that LMC from different allergen sources exhibit disparate barrier regulatory properties, and require further investigation.

Subsequently, we investigated whether BP LMC could potentially induce the repair of a damaged epithelial barrier. Barrier impairment mediated by exposure to papain was significantly counteracted by the addition of BP LMC within three days. However, a similar effect was not observed with simultaneous treatment with BP LMC and papain. This observation may be attributed to the neutralization of their individual effects. Collectively, our data indicate that BP LMC enhance the epithelial barrier integrity even after barrier impairment caused by exposure to an exogenous compound such as papain. However, the possibility of cell detachment at later time points of the experiment, corresponding to the lower CI values, was not validated by visual microscopic inspection. In addition, we quantified the polarized release of mediators after the exposure of 16HBE14o- cells using multiplex immunoassays. More than half of the measured cytokines exhibited a basolateral polarized release at baseline (Supplementary Data, Figure S1). This vectorial apical/basolateral release of cytokines also confirms the polarization of 16HBE14o- cells during exposure to stimuli. Significant modifications were observed upon treatments, especially in the apical compartment (Figure 3). CCL5 is an eosinophil chemotactic factor [23] and TNF-α is a pro-inflammatory cytokine shown to increase ionic permeability in epithelial cells [24]. We observed a significant reduction in apical secretions of CCL5 and TNF-α after treatment with BP LMC when compared to untreated cells, indicating a potential anti-inflammatory property of BP-derived LMC. Cellular signaling proteins such as IL-6, IL-8, IFN-γ and IL-9 have been shown to influence cell permeability by regulating tight junction proteins [25,26,27,28,29]. There was a slight increase in the basolateral levels of IFN-γ and IL-9 after BP LMC exposure. Apical IL-6 and IL-8 levels significantly increased upon treatment with BP extract and slightly decreased when treated with BP LMC. However, the basolateral levels of IL-6 significantly increased upon BP LMC treatment while IL-8 concentrations showed no significant difference (Figure 3 and Supplementary Data, Table S2). This is in contrast to previous studies with grass pollen- and fish-derived LMC that led to an increased release of pro-inflammatory cytokines in both the apical and basolateral compartments [9,10]. The discrepancies between those studies and ours may be attributed to diversity in the composition of LMC derived from different allergen sources, thus exhibiting distinct functions. Limited data are available on the composition of LMC, and therefore it is necessary to characterize the active compounds in LMC from a variety of allergen sources using metabolomics studies.

Modified secretions of GM-CSF, CCL2 and CCL20 following treatment with grass pollen- and fish-derived LMC have been previously demonstrated [9,10]; however, these cytokines were below the detection limit in our experiments.

BP LMC interact with the epithelium independently of the allergens, and their effects remain unchanged in the presence of rBet v 1, as demonstrated in our study. Exposure to the major birch pollen allergen and BP LMC elicit different effects on maintaining barrier integrity and in the vectorial release of mediators. We hypothesize that exposure to BP LMC generates sorting signals that facilitate the enhancement of barrier integrity at the apical side while the basolateral mediators function as intracellular signaling molecules in the orchestration of immune responses. For a comprehensive understanding of the role of BP LMC in allergic sensitization, additional experiments involving primary nasal or bronchial epithelial cells from allergic individuals would be required.

## 4. Materials and Methods

### 4.1. Recombinant Bet v 1 Production and Purification

The sequences coding for recombinant (r) Bet v 1.0101 were inserted into the pET-28b (+) expression vector (Novagen, Darmstadt, Germany) and expressed in *Escherichia coli* NiCo21[DE3] cells (New England Biolabs, Ipswich, MA, USA). Protein expression was induced at 30 °C by the addition of 1 mM of isopropyl β-D-1- thiogalactopyranoside at an OD_600nm_ of 0.8. Cells were then harvested by ultracentrifugation after overnight expression. Cell pellets were frozen for 24 h and lysed by freeze thawing in 50 mM of sodium phosphate buffer, pH 6.5, supplemented with protease inhibitor tablets (Roche Diagnostics, Basel, Switzerland) and 10 mM of dithiothreitol. Purification was achieved through a combination of hydrophobic interaction chromatography (Phenyl Sepharose 6 Fast Flow, GE Healthcare, Uppsala, Sweden), ion exchange chromatography (Q Sepharose Fast Flow, GE Healthcare, Uppsala, Sweden) and gel-filtration chromatography on a Sephacryl S-200 HR column (GE Healthcare, Uppsala, Sweden). Purified rBet v 1 was dialyzed against 10 mM of sodium phosphate buffer, pH 7.4, and Endotoxin Removal Beads (Miltenyi Biotec, Bergisch Gladbach, Germany) were used to remove bacterial endotoxins and DNA.

### 4.2. Birch Pollen Aqueous Extract and Isolation of Low Molecular Weight Components from Birch Pollen

First, 1 g of Commercial *Betula verrucosa* pollen (Allergon, Aengelholm, Sweden) was dissolved in 20 mL of Phosphate-Buffered Saline and gently shaken for 24 h at 4 °C. Birch pollen aqueous extract was obtained by centrifugation, followed by filtering of the supernatant through a 0.45 µm syringe filter. The protein concentration in the aqueous extract was estimated to be 8 mg/mL using the Pierce BCA Protein Assay Kit (Thermo Fisher Scientific, Rockford, IL, USA). BP LMC were isolated from the extract via ultracentrifugation using 3 kDa cut-off filters (Merck Millipore, Darmstadt, Germany) and stored at −20 °C until further use. A single characterized batch was used throughout the experiments.

### 4.3. Bronchial Epithelial Cell Culture

The human bronchial epithelial cell 16HBE14o- (SC150, Sigma-Aldrich, Darmstadt, Germany) was cultured in collagen–fibronectin-coated culture flasks in Minimum Essential Medium (11095080, Gibco, Grand Island, NY, USA) supplemented with 10% fetal bovine serum (10270-106, Gibco, Grand Island, NY, USA), 100 U/mL of penicillin and 100 μg/mL of streptomycin (Sigma-Aldrich, Darmstadt, Germany) in a humidified incubator at 37 °C with a 5% CO_2_ atmosphere. The cells were passaged upon reaching 80% confluency. All the experiments were performed within eight passages.

### 4.4. Cell Viability Assay

First, 16HBE14o- cells were seeded (2.5 × 10^5^ cells/mL) on collagen–fibronectin-coated 96-well plates. Upon forming a tight monolayer, the cells were apically exposed to BP extract (250 µg/mL), rBet v 1 (100 µg/mL), BP LMC (25% *v*/*v*) or a combination of rBet v 1 and BP LMC. These concentrations did not affect cell viability as established in previous experiments through dose-response titration. After 24 h, the cell viability was assessed using an MTT (3-[4,5-dimethylthiazol-2-yl]-2,5 diphenyl tetrazolium bromide) assay as mentioned previously [10]. In brief, the supernatant was removed and 10% MTT (diluted in Tyrode’s salt solution) was added and incubated for 45 min at 37 °C. The formazan crystals were dissolved in 1:6 glycine–dimethyl sulfoxide solution and the absorbance was measured at 565 nm. The percentage of cell viability was calculated by using untreated cells as a reference.

### 4.5. Transepithelial Electrical Resistance Measurement

First, 16HBE14o- cells were seeded (4 × 10^5^ cells/mL) on 12-well permeable filter supports (0.4 μm, 3460, Corning, Kennebunk, ME, USA). Epithelial barrier integrity was assessed periodically by the measurement of transepithelial electrical resistance using an EVOM2 Epithelial Voltohmmeter (World Precision Instruments, Sarasota, FL, USA). The cells were apically exposed to stimuli after the formation of polarized monolayers with a TEER exceeding 1000 Ωcm^2^, and TEER was measured at 3, 6, 12, 18 and 24 h after exposure. The TEER values were normalized to untreated cells at baseline (t = 0 h) and represented as percentage TEER.

### 4.6. Monitoring Epithelial Properties Using xCELLigence System

First, 16HBE14o- cells were seeded (4 × 10^5^ cells/mL) on collagen–fibronectin-coated E-plate 16 (2801032, OLS OMNI Life Science, San Diego, CA, USA) and the transepithelial impedance was monitored using the xCELLigence Real-Time Cell Analysis system (ACEA BioSciences, Santa Clara, CA, USA). The electrical impedance is represented as Cell Index values, which are arbitrary units attributing to cellular characteristics. Once a CI value between 10 and 12 was achieved, which occurs approximately at 16–24 h after cell seeding, the cells were treated. The last measuring time point just before the exposures was selected as the normalization time point, set as t = 0 h, and the corresponding Normalized Cell Index values were calculated relative to this time point. To initiate the exposure of cells at t = 0 h, 50 µL of the medium was removed and replenished by 50 µL containing BP extract (250 µg/mL), rBet v 1 (100 µg/mL), BP LMC (25% *v*/*v*) or a combination of rBet v 1 and BP LMC. The epithelial barrier integrity was monitored for 168 h without any replacement of cell culture medium during this period.

To determine the influence of BP LMC on a damaged barrier, the cells were exposed to papain (70 µg/mL), BP LMC (25% *v*/*v*) or a combination thereof, and epithelial cellular impedance was observed for 216 h (9 days). Papain concentration was selected based on previous experiments in which varying concentrations were tested with the objective to achieve a gradual yet significant decline in Cell Index values over a 96 h period. At 128 h (day 5), to the papain-impaired bronchial epithelial cells, BP LMC (or medium as the control) were added by exchanging 20% of the medium volume, and the CI measurements were continued.

### 4.7. Measurement of Polarized Release of Cytokines

Cell supernatants were collected from the apical and basolateral compartments at 24 h after exposure to BP extract (250 µg/mL), rBet v 1 (100 µg/mL), BP LMC (25% *v*/*v*) or a combination thereof. The Bio-Plex Pro Human Cytokine Screening Panel, Human Inflammation Panel, Human Chemokine Assays and Human Th17 Cytokine Assays (Bio-Rad Lab., Hercules, CA, USA) were used for cytokine quantification. The concentrations of BAFF, CCL2 (MCP-1), CCL5 (RANTES), CCL17 (TARC), CCL20 (MIP-3α), CCL26 (Eotaxin-3), Eotaxin, G-CSF, GM-CSF, IFN-γ, IL-1α, IL-1β, IL-1ra, IL-2, IL-3, IL-4, IL-5, IL-6, IL-7, IL-8, IL-9, IL-10, IL-12 (p40), IL-12 (p70), IL-13, IL-15, IL-16, IL-17A, IL-21, IL-22, IL-25, IL-27 (p28), IL-33, TNF-α, TNF-β, TSLP and VEGF were measured. Fluorescently dyed magnetic beads were pipetted to each well and washed. All washing steps were performed thrice by placing the plate on a magnetic holder. Standards and samples were added to the wells with beads and incubated for 30 min. All incubations were performed in orbital shakers and at room temperature. The plate was washed, after which the detection antibody was pipetted and incubated for 30 min. Next, the plate was washed, and streptavidin–phycoerythrin was added and incubated for 10 min in dark conditions. Finally, assay buffer was added to each well and the plate was measured using the Bio-Plex Manager software version 5.0 on a Luminex xMAP technology instrument. Each cytokine was quantified in relation to the calibration curve (individual for each cytokine).

### 4.8. Statistical Analysis

Statistical analysis was performed using GraphPad Prism version 10.1.0 (GraphPad Software, Boston, MA, USA). Comparisons between treatments were performed using one-way ANOVA followed by Tukey’s multiple comparison test. The significances are represented in the plot: * indicates *p* < 0.05, ** *p* < 0.01, *** *p* < 0.001, **** *p* < 0.0001. The graphs in all the figures were created using GraphPad Prism version 10.1.0 (GraphPad Software, Boston, MA, USA).

## Data Availability

The data generated and analyzed during the current study are available from the corresponding author on reasonable request.

## Supplementary Materials

The following supporting information can be downloaded at: https://www.mdpi.com/article/10.3390/ijms25137374/s1.
