# Supplementary material for: Modulation of Bronchial Epithelial Barrier Integrity by Low Molecular Weight Components from Birch Pollen
Source: Int J Mol Sci. 2024 Jul 5;25(13):7374. doi: 10.3390/ijms25137374 (PMC11242533; doi:10.3390/ijms25137374)
Supplement: Supplementary file 1 [file ijms-25-07374-s001.zip › ijms-3047332-supplementary.pdf]

Supporting material

Table S1. Statistical analysis of Normalized Cell Index values. Adjusted *P* values of the correlations between the Normalized Cell Index values after treatment with papain, LMC or their concurrent treatment (as shown in Figure 2). The comparisons were performed using One-way ANOVA with Tukey's multiple comparison test. . $*p < 0.05$, $**p < 0.01$, $***p < 0.001$, $****p < 0.0001$.

Hours	Comparisons	Summary	Adjusted *p* value
24	Untreated cells vs. Papain	**	0.0032
	Untreated cells vs. BP LMC	ns	0.6667
	Untreated cells vs. Papain + LMC	**	0.0034
	Papain vs. BP LMC	*	0.0403
	Papain vs. Papain + LMC	ns	>0.9999
	BP LMC vs. Papain + LMC	*	0.0424
48	Untreated cells vs. Papain	*	0.0255
	Untreated cells vs. BP LMC	ns	0.6065
	Untreated cells vs. Papain + LMC	ns	0.1309
	Papain vs. BP LMC	ns	0.2697
	Papain vs. Papain + LMC	ns	0.8453
	BP LMC vs. Papain + LMC	ns	0.7184
72	Untreated cells vs. Papain	ns	0.1118
	Untreated cells vs. BP LMC	ns	0.9987
	Untreated cells vs. Papain + LMC	ns	0.8905
	Papain vs. BP LMC	ns	0.148
	Papain vs. Papain + LMC	ns	0.3624
	BP LMC vs. Papain + LMC	ns	0.9427
96	Untreated cells vs. Papain	ns	0.1107
	Untreated cells vs. BP LMC	ns	0.7762
	Untreated cells vs. Papain + LMC	ns	>0.9999
	Papain vs. BP LMC	*	0.0156
	Papain vs. Papain + LMC	ns	0.1016
	BP LMC vs. Papain + LMC	ns	0.8004
120	Untreated cells vs. Papain	**	0.0052
	Untreated cells vs. BP LMC	ns	0.3797
	Untreated cells vs. Papain + LMC	ns	0.968
	Papain vs. BP LMC	***	0.0001
	Papain vs. Papain + LMC	*	0.0143
	BP LMC vs. Papain + LMC	ns	0.1888
144	Untreated cells vs. Papain	****	<0.0001
	Untreated cells vs. BP LMC	**	0.0055
	Untreated cells vs. Papain + LMC	ns	0.0765
	Papain vs. BP LMC	****	<0.0001

	Papain vs. Papain + LMC	***	0.0005
	BP LMC vs. Papain + LMC	****	<0.0001
168	Untreated cells vs. Papain	****	<0.0001
	Untreated cells vs. BP LMC	****	<0.0001
	Untreated cells vs. Papain + LMC	ns	0.0642
	Papain vs. BP LMC	****	<0.0001
	Papain vs. Papain + LMC	***	0.0005
	BP LMC vs. Papain + LMC	****	<0.0001
192	Untreated cells vs. Papain	ns	0.0515
	Untreated cells vs. BP LMC	****	<0.0001
	Untreated cells vs. Papain + LMC	ns	0.8185
	Papain vs. BP LMC	****	<0.0001
	Papain vs. Papain + LMC	**	0.0078
	BP LMC vs. Papain + LMC	****	<0.0001
216	Untreated cells vs. Papain	ns	0.8714
	Untreated cells vs. BP LMC	****	<0.0001
	Untreated cells vs. Papain + LMC	ns	0.3204
	Papain vs. BP LMC	****	<0.0001
	Papain vs. Papain + LMC	ns	0.087
	BP LMC vs. Papain + LMC	****	<0.0001

BP, birch pollen; LMC, low molecular weight components; ns, not significant.

Table S2. Cytokine concentrations. Concentrations of individual cytokines released after 24 hours of treatment with BP extract, rBet v 1, BP LMC or a combination thereof, in the apical and basolateral compartments listed as pg/mL units.

	**Mediator**	**Untreated cells**	**BP Extract**	**rBet v 1**	**BP LMC**	**rBet v 1 + BP LMC**
Apical secretion	CCL2 (MCP-1)	3.02	3.59	4.20	4.35	3.80
	CCL20 (MIP-3α)	101.16	112.14	119.23	113.97	129.38
	CCL26 (Eotaxin-3)	12.30	9.72	9.69	7.13	5.38
	CCL5 (RANTES)	10046.38	11466.13	11999.08	3543.03	5228.03
	Eotaxin	0.21	0.19	0.14	0.19	0.17
	G-CSF	61.38	77.70	55.16	31.12	36.64
	GM-CSF	2.79	3.72	3.58	2.36	3.01
	IFN-γ	5.99	5.62	3.84	6.07	4.42
	IL-16	0.96	0.96	1.09	1.97	
	IL-17a	1.13	2.32	1.04	2.25	1.93
	IL-1B	0.48	0.29	0.40	0.29	0.57
	IL-1ra	146.96	119.78	77.77	86.36	88.48
	IL-25	0.32	0.11	0.22	0.62	0.15
	IL-27 p28	0.32	0.11	0.22	0.62	0.15
	IL-6	1439.36	1843.77	1647.91	1225.28	1369.71
	IL-7	4.71	4.70	2.47	2.45	2.32
	IL-8	2024.15	2541.39	2678.89	1626.84	1617.85
	IL-9	2.89	2.38	2.57	2.02	2.94
	TARC	1.94	2.20	2.74	2.52	2.52
	TNF-a	1988.22	2570.02	2484.91	1231.82	1431.90
	TNF-b	11.52	15.26	13.84	8.72	10.64
	VEGF	313.94	386.17	324.38	192.96	285.83
Basolateral secretion	CCL2 (MCP-1)	2.19	2.43	1.71	3.01	2.50
	CCL20 (MIP-3α)	82.53	57.87	45.95	63.79	75.84
	CCL26 (Eotaxin-3)	4.46	4.67		4.04	4.73
	CCL5 (RANTES)	1630.26	1655.19	1675.67	1544.56	1558.45
	Eotaxin	0.16	0.22	0.14	0.17	0.23
	G-CSF	35.23	34.88	33.68	45.99	40.86
	GM-CSF	0.77	0.79	0.78	1.00	0.94
	IFN-γ		3.73	2.22	1.94	6.42
	IL-16	1.47	1.21	1.21		0.96
	IL-17a	1.14	1.00	0.41	1.46	0.27
	IL-1B		0.38	2.22	0.29	0.32
	IL-1ra	4.58	11.11		12.66	8.12
	IL-25	0.32	0.18	0.12	0.28	0.20
	IL-27 p28	0.32	0.18	0.12	0.28	0.20
	IL-6	554.37	629.56	550.15	684.55	598.13
	IL-7	0.44	1.72	1.06	0.11	0.61
	IL-8	1583.68	1636.38	1427.20	1698.97	1493.04
	IL-9	0.43	1.54	1.11	3.84	3.03

	TARC	1.59	0.90	0.53	1.32	1.03
	TNF-a	435.54	514.51	489.57	438.75	419.60
	TNF-b	2.34	2.68	2.13	2.11	2.51
	VEGF	395.50	390.62	398.62	554.65	446.73

BP, birch pollen; CCL, CC chemokine ligand; G-CSF, granulocyte-colony stimulating factor; IL, interleukin; TNF, tumor necrosis factor; VEGF, vascular endothelial growth factor. Blank values represent values below the detection limit. The values represent the mean of three experiments.

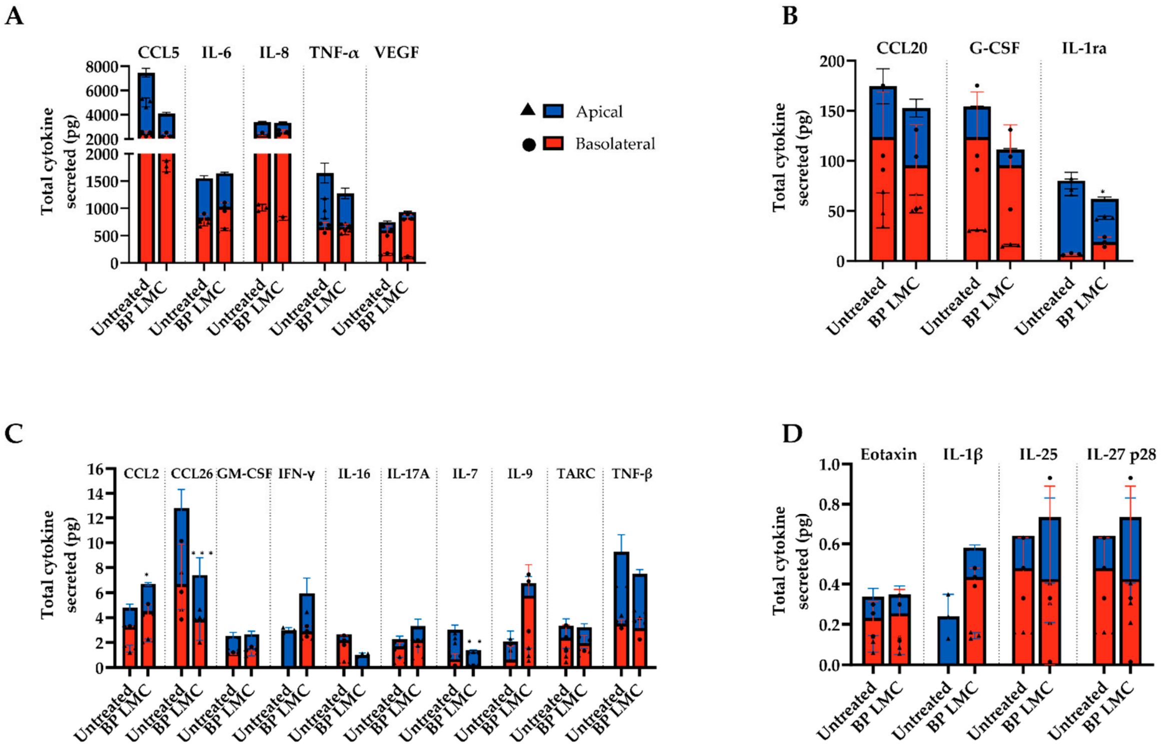

Figure S1. Total cytokine amounts secreted after 24 h of exposure to BP LMC. Apical and basolateral supernatants were collected 24 hours after exposure of the polarized 16HBE14o- cells and the cytokines were quantified. Triangle and circle symbols represent the data points of apical and basolateral values respectively. Graphs depict mean cytokine concentrations ± SD from three different experiments and asterisks indicate a statistically significant difference in total cytokine secretion compared to the untreated cells. *$p < 0.05$, **$p < 0.01$, ***$p < 0.001$.
